# Into the Wild: *Oryza* Species as Sources for Enhanced Nutrient Accumulation and Metal Tolerance in Rice

**DOI:** 10.3389/fpls.2016.00974

**Published:** 2016-06-29

**Authors:** Felipe K. Ricachenevsky, Raul A. Sperotto

**Affiliations:** ^1^Departamento de Biologia, Programa de Pós-Graduação em Agrobiologia, Universidade Federal de Santa MariaSanta Maria, Brazil; ^2^Setor de Genética e Biologia Molecular do Museu de Ciências Naturais, Programa de Pós-Graduação em Biotecnologia, Centro de Ciências Biológicas e da Saúde, Centro Universitário UNIVATESLajeado, Brazil

**Keywords:** metal tolerance, natural variation, nutrient use efficiency, rice domestication, wild rice species

## Rice and *Oryza* genus diversity

Rice (*Oryza sativa* L.) is one of the most important crops in the world (Elert, 2014). However, rice grain production can be affected by nutritional status, depending on the cultivation method and soil type: high levels of metals such as aluminum (Al) and iron (Fe) decrease plant growth and yield, especially in acidic soils (Ricachenevsky et al., 2010; Kochian et al., 2015), while low nutrient use efficiency leads to increased fertilization needs (Koutroubas and Ntanos, 2003). Rice grains, which are key staple food for more than half of the world's population, can accumulate toxic elements such as arsenic (As), or show low concentration of key nutrients to human nutrition, such as Fe and zinc (Zn) (Sperotto et al., 2012; Ricachenevsky et al., 2015; Clemens and Ma, 2016). Thus, attempts to improve metal tolerance, nutrient use efficiency, and nutrient accumulation in grains are likely to have an impact on both agriculture and rice grains nutritional value for consumption.

Cultivated rice (*O. sativa*) is part of the *Oryza* genus, which is composed of 23 species, including the cultivated African rice *Oryza glaberrima*, plus 21 wild relatives (Jacquemin et al., 2013). These species show 11 different genome types (AA, BB, CC, BBCC, CCDD, EE, FF, GG, KKLL, HHJJ, HHKK; Lu et al., 2009; Jacquemin et al., 2013; Atwell et al., 2014), and have a pan-tropical distribution, growing in a broad range of environments (Atwell et al., 2014). Since *O. sativa* was domesticated from a limited number of *O. rufipogon* genotypes, its closest wild relative, it is estimated that only 10–20% of the genetic diversity found in wild species is present in cultivated rice germplasm (Zhu et al., 2007; Palmgren et al., 2014). Although many efforts were made to find natural variation within the rice germplasm that could improve nutrition-related traits in cultivated rice, the narrow genetic diversity can be a limiting factor (Li et al., 2014; Yan et al., 2016). Considering the diversity of rice wild species and their distinct growing environments (Atwell et al., 2014), we can expect that they will be adapted to different nutrient availabilities. Thus, wild relatives are a potential source of interesting alleles or even new mechanisms of metal and metalloid accumulation control. However, these genetic resources are almost unexplored, with very few studies screening these species for interesting phenotypes, especially for metal-related traits.

## Enhanced nutrient accumulation and use efficiency

A lot of progress to understand the molecular and physiological mechanisms that control specific steps of the nutrient homeostasis network has been done in the last years, leading to improved nutrient accumulation and use efficiency in crop plants. However, our basic knowledge on metal homeostasis is far from complete, and its understanding is an urgent step needed to sufficiently enhance plant mineral acquisition and storage (Ghandilyan et al., 2006).

Quantitative Trait Loci (QTL) analysis is a powerful approach to detect chromosomal regions affecting quantitative traits, and several QTL mapping have been conducted for rice grain nutrients accumulation. Using crosses of *O. sativa* × *O. rufipogon*, Garcia-Oliveira et al. (2009) and Hu et al. (2016) were able to detect numerous QTLs for different mineral nutrients accumulation in rice grains, and wild rice (*O. rufipogon*) contributed with favorable alleles for most of the QTLs. It is important to highlight that both authors found co-locations of QTLs for some minerals, suggesting that the same allele could be used to simultaneously improve rice grains nutritional quality. These QTLs are good candidates for fine-mapping and cloning of candidate genes.

Studies comparing wild and cultivated rice responses to nutrient deficiencies or use efficiencies are scarce. Chiangmai and Yodmingkhwan (2010) compared the responses of wild and cultivated rice varieties to phosphate (Pi) application, and found that *O. rufipogon* have greater response to Pi-application than cultivated rice varieties. The positive aspect of *O. rufipogon* is that it may be used as germplasm for improving the cultivated rice varieties responses to P-application. On the other hand, the F1 hybrid of the *O. rufipogon* and cultivated rice varieties may be more adaptive to the environment and more competitive in the rice field, being more difficult to eradicate (Chiangmai and Yodmingkhwan, 2010). It would be interesting to test the interaction of two factors: nutrient application (in different concentrations) and rice genotypes, including cultivated and wild species. This would help to compare the response of rice varieties to nutrient application more effectively.

Cultivated rice (*O. sativa*) accumulates high concentration of silicon (Si), which is required for its high and sustainable production. Mitani-Ueno et al. (2014) showed that Si uptake was lower in *O. rufipogon, O. barthii, O. australiensis*, and *O. punctata*, compared to the cultivated rice, *O. sativa*. However, when grown in the field, all of them showed similar concentration of Si in the shoots. Therefore, Si uptake ability by the roots is different within wild rice species and cultivated rice, but the similar shoot Si concentration suggests that wild rice has an ability to optimize Si accumulation in the shoots, which is important for healthy plant growth and production (Mitani-Ueno et al., 2014). According to the authors, high Si accumulation in cultivated rice is achieved by a high expression of both influx (Lsi1) and efflux (Lsi2) Si transporters in roots. As wild rice species can optimize Si accumulation in the shoots, even with lower mRNA expression of *Lsi1* and *Lsi2* genes, it would be interesting to find other molecular players that control Si homeostasis in Si-efficient wild rice species. Since Lsi1 and Lsi2 transporters are also involved in As transport, a toxic, non-essential element that is also efficiently absorbed into rice plants, including grains (Clemens and Ma, 2016), the use of distinct alleles for these transporters (with different expression levels or altered affinities) from wild relative might be helpful to fine-tune Si/As uptake and distribution.

## Natural variation and metal tolerance

Aluminum (Al^3+^) toxicity is a widespread problem for crops growing in acidic soils, which compose around 40% of the arable land (Uexkuïll and Mutert, 1995). At low pH, Al^3+^ becomes highly available, severely decreasing root growth and leading to nutritional deficiencies and drought. Plants show Al avoidance mechanisms, which involves organic acid secretion to the rhizosphere and Al^3+^ chelation; and Al tolerance, in which Al^3+^ is detoxified inside the root vacuoles, avoiding toxicity to the cell wall (Kochian et al., 2015).

Rice is a model for Al-tolerant crops, since it developed both avoidance and tolerance mechanisms. NRAT1 (Nramp aluminum transporter) is a unique plasma membrane-localized transporter through which Al^3+^ enters the root symplast, working in concert with vacuolar transporters to detoxify Al^3+^ into the vacuoles (Kochian et al., 2015). Interestingly, NRAT1 locus was recently implicated in natural variation of Al tolerance in rice: tolerant rice accessions showed higher expression of NRAT1 than sensitive ones, and some sensitive accessions carry missense mutations in NRAT1 coding sequence (Li et al., 2014).

*Oryza rufipogon*, the common wild rice, is known as an Al tolerance species, with higher tolerance than cultivated rice, possibly even stimulating root elongation depending on Al^3+^ concentration (Nguyen et al., 2003; Cao et al., 2011). Crosses of *O. rufipogon* × *O. sativa* were used to map many QTLs for Al stress tolerance (Nguyen et al., 2003). Interestingly, the major QTL, which explained 24.9% of the variation, is located in chromosome 3. NRAT1, associated with Al tolerance in cultivated rice germplasm, sits on chromosome 2, indicating that the major QTL from *O. rufipogon* is not NRAT1, and thus it would be possible to pyramid these tolerance genes. Moreover, other wild relatives are virtually unexplored for their Al tolerance, although they can be found in soils with high Al^3+^ levels (Vianello Brondani et al., 2005).

Iron toxicity is another well-known problem for rice production, especially for lowland rice. Upland rice is grown under non-flooded, aerobic conditions, which keeps Fe in its oxidized form (Fe^3+^) that binds to soil particles, with low availability for uptake. However, under flooded conditions, Fe becomes highly available as Fe^2+^, leading to Fe overload and consequent toxicity (Ricachenevsky et al., 2010). Although there are rice cultivars with relative Fe tolerance, there is still need for improvement. In Africa, up to 60% of the rice area in West African countries is affected by this stress, which results in yield losses of 50% (Audebert and Fofana, 2009). Interestingly, the other cultivated species of rice, African rice *O. glaberrima*, showed superior tolerance to Fe toxicity when compared to *O. sativa*, and thus could potentially be used to breed Fe excess tolerance into *O. sativa* cultivars. In a large screen, several accessions of *O. glaberrima* from the AfricaRice gene bank where tested for grain yield in the field under control and Fe toxicity conditions, and compared to *O. sativa* controls. Three accessions were found to have higher yield than *O. sativa* cultivars under stress, but comparable yield in control condition, and thus could be used in breeding programs (Sikirou et al., in press). Another recent study found seven QTLs for Fe tolerance in crosses of *O. glaberrima* × *O. sativa*, with *O. glaberrima* contributing with the tolerance alleles (Dufey et al., 2015).

## Future perspectives

Rice domestication was performed with a narrow genetic diversity, and less than 20% of wild species diversity is present in cultivated rice (Palmgren et al., 2014). Therefore, searching for variability in wild rice has great potential for rice breeding, since it might have important genes for enhanced nutrient accumulation and metal tolerance, already lost in cultivated rice. Even close relatives such as *O. rufipogon* or *O. glaberrima* present useful traits that are not found within *O. sativa* diversity. Breeding varieties that have improved nutrient use efficiency and accumulation depends on large genetic variation for root uptake, root-to-shoot transport, xylem and phloem loading and unloading, mineral remobilization/compartmentalization, and grain accumulation. Also, minimum requirements for nutrients must be fulfilled while simultaneously avoiding their possible toxic effects, as well as other toxic elements (Ghandilyan et al., 2006). This is not an easy task, since many transporters are not specific, being able to transport different nutrients with different specificities, including toxic ones. Then, in future genetic modification projects, we need to know which specific genes should be targeted, and also emphasize the expression in specific tissues and stages, instead of using constitutive expression. Natural variation in transporter genes might indicate important residues for selectivity, and useful sequences might be further improved by site-directed mutagenesis (Menguer et al., 2013; Yan et al., 2016). At the same time, search for genotypes with low concentration of anti-nutrients such as phytate might be fruitful (Ghandilyan et al., 2006).

Several genes that encode proteins important to mineral homeostasis, including membrane transporters, storage proteins, metal ligands with different substrate specificities, receptors and regulatory proteins have been identified in rice (Sperotto et al., 2009; Ricachenevsky et al., 2015). The knowledge accumulated will be key for fast characterization of protein involved in uptake and transport of metals in wild relatives, using comparative transcriptomics and functional characterization of orthologous genes (Mitani-Ueno et al., 2014). QTL mapping of traits in wild relatives should be explored, and introgression of desirable traits by crossing with *O. sativa* is feasible (Jena, 1994; Collard and Mackill, 2008). Genome-wide association study (GWAS), which has been successfully used to map complex traits in rice (Kumar et al., 2015), has great potential for identifying the underlying genes causing natural variation in nutrient accumulation and metal tolerance (Norton et al., 2014; Huang and Salt, 2016; McCouch et al., 2016).

Yet, to identify the available genetic variation, extensive screening studies on wild rice species accessions is the first step. As wild rice species are distributed across several biomes worldwide (Atwell et al., 2014), different research groups need to join efforts to uncover potentially useful traits. In order to facilitate the access to wild rice material and information, databases on molecular and physiological characteristics should be developed, likely in association with seed distribution centers such as Genesys (https://www.genesys-pgr.org/). Already existing platforms such as the IonomicsHub (www.ionomicshub.org; Baxter et al., 2007) might be a useful starting point. Although the use of crop wild relatives is still in its beginnings, the few studies available clearly indicate that we should move forward to unlock the full potential of these species for enhanced nutrient accumulation and metal tolerance.

## Author contributions

Both authors have made substantial, direct and intellectual contribution to the work, and approved it for publication.

### Conflict of interest statement

The authors declare that the research was conducted in the absence of any commercial or financial relationships that could be construed as a potential conflict of interest.
